# Evaluation of Acute Outcomes and Factors Influencing the Care of Chest Trauma in a District General Hospital in the United Kingdom

**DOI:** 10.7759/cureus.45690

**Published:** 2023-09-21

**Authors:** Gur Aziz Singh Sidhu, Aveen Mahmood, Saphalya Pattnaik, Mohammed Subratty, Harjot Kaur, Venkataraman Raja, Shyam Rajagopalan, Neil Ashwood

**Affiliations:** 1 Orthopedics, University Hospital Lewisham, London, GBR; 2 Trauma and Orthopaedics, University Hospitals of Derby and Burton NHS Foundation Trust, Burton-on-Trent, GBR; 3 Orthopedics and Traumatology, Max Super Speciality Hospital, Delhi, IND; 4 Trauma and Orthopaedics, Queen's Hospital Burton, Burton-on-Trent, GBR; 5 Trauma and Orthopaedics, University Hospital Lewisham, London, GBR; 6 Anaesthesia, Queen Elizabeth Hospital, London, GBR; 7 Trauma and Orthopaedics, Research Institute of Healthcare Sciences, University of Wolverhampton, Wolverhampton, GBR

**Keywords:** post-traumatic haemothorax, life-threatening events, rib fractures, polytrauma patient, chest wall trauma

## Abstract

Background

The rate of chest trauma admissions under the Queen Hospital Burton Orthopedic team has been steadily increasing, surpassing other hospital trusts. Patients are managed locally by the Orthopedic department, unlike in major trauma centres. Understanding the management outcomes and patient factors in this setting is crucial for enhancing patient safety.

Methodology

A retrospective analysis of 139 patients with chest trauma referred to the QHB Orthopedic team from October 2017 to May 2021 was conducted using the Meditech-V6 electronic medical records system (Meditech, Westwood, US). This study aims to evaluate the outcomes of patients admitted with chest trauma and improve current practices. The objectives include assessing patient factors influencing outcomes, initiating discussions with a major trauma centre, and enhancing the quality of care for chest trauma patients.

Results

The mechanism of injury in all cases of chest injuries was blunt trauma, accounting for 100% of the cases. The specific mechanisms of injury observed in the study included falls from standing, falls from height, road traffic collisions, and assault. The study comprised 139 individuals, 128 of whom were diagnosed with rib fractures, and 11 who did not have any rib fractures. In addition, two patients were hospitalized with bilateral rib fractures, both of which were life-threatening. Tragically, one of these cases resulted in the death of the patient. With regard to outcomes, 67% of the patients received a consultation at Royal Stoke Hospital (RSH). Eight individuals were transferred to RSH for further management, while the remaining 131 patients were not transferred. Eighty-seven individuals were discharged from the hospital, indicating successful recovery and readiness for discharge. However, it is noteworthy that nine patients experienced complications during their hospital stay, highlighting the potential challenges and risks associated with chest trauma management. Tragically, seven patients succumbed to their injuries and passed away.

Conclusions

The majority of patients in this study were aged 65 and over and presented with multiple comorbidities, indicating the complex medical profile of this population. However, despite the presence of life-threatening injuries and the associated risks, only a minority of patients in the study were transferred to a designated trauma centre. This raises concerns about the adequacy of the current transfer protocols and the potential impact on patient outcomes.

## Introduction

Thoracic trauma is a common presentation in accident and emergency (A&E) departments worldwide [1]. Rib fractures often lead to immediate damage to the underlying internal organs which could lead to subtle to life-threatening complications including haemothorax, pneumothorax, and cardiac contusions. Notably, the data from the Trauma Audit Research Network (TARN) in 2017 revealed that the thorax is the second most affected anatomical region among individuals aged 60 years and above [2]. It is estimated that approximately 20% of all cases of thoracic trauma are associated with concurrent rib fractures [3]. Despite a growing body of evidence highlighting the significant impact of rib fractures on morbidity and mortality, particularly in the elderly population, there is currently a lack of national guidelines for the management of such injuries [1,4].

The trends in chest trauma admissions under the Orthopaedic team in our hospital have been rapidly rising since 2017, with an average of 30 admissions per year. The analysis reveals a significant increase in admissions over the past four years, culminating in the highest number of admissions recorded in 2021 (total: 41 admissions). In contrast to other hospital trusts, where chest trauma cases are typically managed by the Thoracic team at major trauma centres (e.g., Royal Stoke Hospital/ Queens Medical Centre Nottingham), patients are admitted for local conservative management under the Orthopedic department and the chest trauma pathway is followed for their management.

## Materials and methods

**Figure 1:**
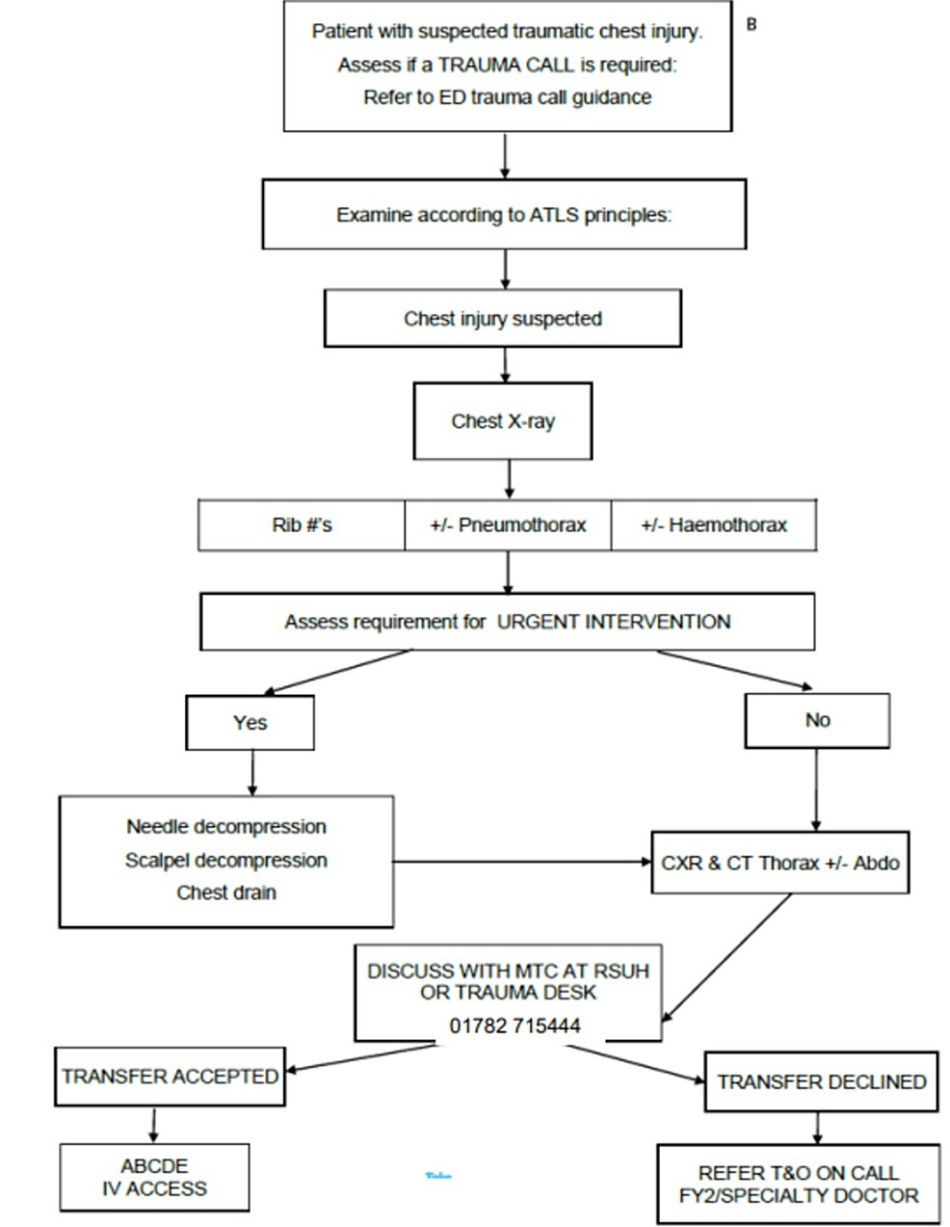
Pathway for chest trauma patients in our hospital (taken after due permission from the Accident & Emergency Department of QHB) ED: Emergency department; ATLS: adult trauma life support; CXR: chest X-ray; CT: computed tomography; MTC: major trauma centre; RSUH/RSH: Royal Stokes University Hospital/Royal Stokes Hospital; T &O: Trauma and Orthopaedics; FY 2: foundation year 2; ABCDE: airway, breathing, circulation, disability, exposure; IV: intravenous

This retrospective study aimed to analyze the patient population with chest trauma referred to the Orthopedic team from the A&E department at Queens Hospital Burton, United Kingdom from October 2017 to May 2021. The inclusion criteria involved patients aged from 20 to 100 years who presented to the A&E department with chest trauma. The pathway for chest trauma our trust involved patients being admitted under orthopaedics for further care if this needed admission (Figure 1). The patients were discussed with the level I trauma care centre hospital for further treatment plans. Patients were shifted to the level I trauma centre who were deemed fit for surgical intervention or life-threatening situations. Patients with chest trauma were categorized into two groups based on the number of comorbidities they had: those with fewer than three and those with three or more. The patients with associated head injuries were excluded from this study.

The aim of this study was to review patients admitted with chest trauma and their management outcomes, in view of updating our practice to improve patient safety. The main objective was to look at patient factors (number of rib fractures or comorbidities) that influence their outcome, the discussion with the major trauma centre, and ultimately the outcome from their care. The findings of this study provide valuable insights into the characteristics and management of chest trauma patients, which can contribute to improved clinical decision-making and patient care in similar healthcare settings. The data collection was conducted using the Meditech-V6 electronic medical records system (Meditech, Westwood, Massachusetts, USA). The statistical significance was recorded with p-value <0.005.

## Results

The study comprised 139 individuals in total, 128 of whom were diagnosed with rib fractures, and 11 who did not have any rib fractures but other injuries. In addition, two patients were hospitalized with bilateral rib fractures, both of which were life-threatening. Tragically, one of these cases resulted in the death of the patient. The mechanism of injury in all cases (100%) of chest injuries was blunt trauma. The specific mechanisms of injury observed in the study included falls from standing, falls from height, road traffic collisions, and assault.

According to the data collected from 139 patients, the age range for chest trauma cases varied significantly. The oldest patient observed was 98 years old, while the youngest patient affected was 21 years old. The median age of patients suffering from chest trauma was around 69 years. The age distribution of patients in our cohort has been presented in Table 1.

**Table 1:** Age distribution of patients in our cohort

Age group (Years)	Number of patients
20-40	19
40-60	31
60-80	38
80-100	51

The overall analysis of gender distribution among chest trauma patients revealed a higher number of males compared to females. Of the total patients, 86 (62%) were males, while 53 (38%) were females. The minimum length of stay observed for any patient was 0 days, suggesting instances where patients might have received outpatient care. On the other hand, the maximum length of stay recorded was 52 days, indicating cases of intricate chest trauma necessitating an extended hospital stay (Table 2). 

**Table 2:** Duration of stay in our cohort

Duration of stay (days)	Number of patients (n)
Day 0	3
Day 1-10	100
Day 11-20	21
Day 21-30	9
Day >30	6

The number of rib fractures had a notable effect on the length of patient stay, as those with a higher number of rib fractures experienced longer stays. Specifically, patients without any rib fractures had an average stay duration of two days, while patients with less than three rib fractures or three to five rib fractures had an average stay duration of five days. Conversely, patients with more than six rib fractures had the lengthiest average stay duration, determined to be 8.5 days. The analysis of rib fractures in the patient population revealed varying frequencies across different categories. The majority of patients (93%) had three to six rib fractures. Only eight patients (6% of the patients) suffered more than six rib fractures. Other chest injuries were detected in the patient group, in addition to rib fractures including pneumothorax, haemothorax, flail chest, and sternal fractures. In our study, 97 individuals (72% of the sample) were found to have had such life-threatening injuries (Table 3). 

**Table 3:** Life-threatening injuries in our cohort

Life-threatening injuries	Number of patients (percentage)
Haemothorax	22 (16%)
Pneumothorax	25 (18%)
Flail chest	10 (7.19%)
Haemopneumothorax	17 (12.2%)
Parenchymal injury	23 (16.5%)

A total of 21 out of 139 patients included in the study underwent the insertion of a chest drain as part of their management and rest were managed symptomatically. Notably, all 21 patients with a chest drain had life-threatening injuries, indicating the severity of their condition. The average length of stay for patients who received a chest drain was found to be nine days. 

The presence of multiple comorbidities was found to be associated with a longer duration of patient stay. Among the patients under the age of 65, 48 individuals (<3 comorbidities) had an average stay of four days and 10 individuals (>3 comorbidities), the average stay extended to seven days. The average stay for people over 65 years was nine days (<3 comorbidities) and 13 days (>3 comorbidities) highlighting the impact of age and multiple comorbidities on the hospitalization length.

The patient outcomes in the study were analyzed, revealing that 67% of the patients received a consultation at Royal Stoke Hospital (RSH). Out of the total patient population, eight individuals were transferred to RSH in view of life-threatening injuries for further management, while the remaining 131 patients were not transferred. Among the patients included in the study, 87 individuals were discharged from the hospital, indicating successful recovery and readiness for discharge. However, it is noteworthy that nine patients experienced complications during their hospital stay, highlighting the potential challenges and risks associated with chest trauma management (Table 4). Tragically, seven patients succumbed to their injuries and passed away due to either pneumonia, COVID-19, or congestive cardiac failure.

**Table 4:** Mortality in our cohort T2DM: Type 2 diabetes mellitus; HTN: hypertension; CABG: coronary artery bypass grafting; IHD: ischemic heart disease; PMR: polymyalgia rheumatica; OA: osteoarthritis; CVA: cerebrovascular accident; SDH: subdural hemorrhage; AF: atrial fibrillation; CCF: congestive cardiac failure; COPD: chronic obstructive pulmonary disease

Age (at time)	Sex	Duration of Stay (days)	Side of Injury	Number of Ribs Broken	Chest Injury	Chest Drain Inserted	Associated Injuries	Medical Comorbidities	Chest injury contributing factor?
88	M	8	R	6	Flail chest	Yes	No	Dementia, T2DM,	No
84	F	10	Bilateral	5	-	No	Yes (fracture)	Dementia, HTN	No
82	F	13	L	2	Pneumothorax	No	Yes (fracture)	Dementia, HTN	No
84	M	15	R	5	-	No	No	Dementia, T2DM, HTN, CABG, IHD	Yes
84	F	5	R	6	Haemothorax, effusions	No	Yes (fracture)	PMR, OA, CVA, anaemia, previous SDH, HTN	No
85	F	17	R	7	Haemopneumothorax, flail segment	Yes	Yes (fracture)	AF, asthma, OA, CCF	Yes
61	M	6	R	5	pneumothorax	Yes	No	COPD, alcohol dependanceT2dm	No

## Discussion

It is noteworthy to highlight that individuals aged 65 and above represent a substantial portion, exceeding 40% of the population affected by rib fractures [5]. This demographic information holds significance as previous studies have demonstrated a positive association between age and the incidence of complications linked to rib fractures, including pneumonia and the need for intensive care unit admission [6,7]. In a study conducted by Bulger et al., it was revealed that older adults aged 65 and above experienced twice the mortality and morbidity rates when hospitalized for rib fractures compared to younger counterparts [5]. On the contrary, our study reported that the risk of developing a life-threatening injury was found to be similar across both age categories, indicating that age did not significantly impact the likelihood of sustaining such injuries. Furthermore, patients with no rib fractures had a 27% association with life-threatening injuries, whereas those with less than three rib fractures had a 48% association. The association increased to 53% for patients with three to six rib fractures and significantly higher to 88% for patients with more than six rib fractures. 

Another interesting observation in our study was that the average length of stay for patients who received a chest drain was found to be nine days, which was higher than the general average length of stay observed in the study of 4.5 days. Moreover, the number of comorbidities did affect their length of stay in the hospital highlighting the impact of multiple comorbidities on hospitalization length.

Further analysis of life-threatening injuries concerning the number of rib fractures revealed a positive association between a higher number of rib fractures and the occurrence of life-threatening injuries (p<0.005). Specifically, patients with no rib fractures had a 27% association with life-threatening injuries, whereas those with less than 3 rib fractures had a 48% association. The association increased to 53% for patients with three to six rib fractures and significantly higher to 88% for patients with more than six rib fractures. Furthermore, the risk of developing a life-threatening injury was found to be similar across both age categories, indicating that age did not significantly impact the likelihood of sustaining such injuries.

Interestingly, there was no significant increase in the average length of stay for patients with life-threatening injuries, which remained at approximately 8-9 days. However, when examining patients with more than six rib fractures, a higher average duration of stay of 12 days was observed compared to patients with fewer rib fractures, who had an average stay duration of seven days. This discrepancy suggests the complexity and severity of cases involving more than six rib fractures, necessitating additional care and resources during hospitalization. It is noteworthy that despite the absence of a general increase in the length of stay for patients with life-threatening injuries, the extended stay for those with more than six rib fractures underscores the unique challenges presented by such situations.

The transfer of patients to RSH exhibited varying degrees of delay. Among the patients requiring transfer, one patient was promptly transferred, while three patients were transferred within a day. In contrast, three patients experienced delays of more than two days before being transferred to RSH. The reasons for these delays were attributed to delayed discussions with RSH in one case and limited bed availability at RSH for the remaining two cases. Similarly, in the context of transfers to other trauma centres, three patients were transferred to QMC Nottingham, while one patient was transferred to Derby Hospital. Among the patients transferred to QMC Nottingham, two encountered delays in the transfer process. Specifically, one patient experienced a delay of 1-2 days, while the other patient faced a delay of more than two days.

This study examines the causes of death and the presence of chest injury as a contributing factor among a group of seven deceased patients. The analysis revealed a range of causes for these unfortunate fatalities. Among the patients, 14% (n=1) died as a result of aspiration pneumonia and congestive heart failure, while an additional 14% (n=1) succumbed to fluid overload and hospital-acquired pneumonia (HAP). COVID-19 pneumonia was identified as the cause of death for another patient, also accounting for 14% (n=1) of the cases. HAP alone accounted for 14% (n=1) of the deaths, whereas a combination of HAP and COVID-19 pneumonia led to the demise of 29% (n=2) of the patients. Moreover, 14% (n=1) of the patients experienced an exacerbation of chronic obstructive pulmonary disease in conjunction with HAP. In terms of chest injury as a contributing factor, 43% (n=3) of the deceased patients had it listed, while the remaining 57% (n=4) did not have chest injury listed as a contributing factor to their death. These findings shed light on the specific causes of death among the patients in the study, highlighting the importance of recognizing and addressing complications related to chest trauma.

Moreover, rib fractures contribute significantly to the caseload of general surgical teams in district general hospitals (DGHs). Additionally, chest injuries account for approximately 25% of trauma-related fatalities and are associated with considerable morbidity consequences. Survivors of chest injuries often experience prolonged absence from work and diminished functional capacity [8,9].

We had some limitations for this study. Firstly, the study design employed a retrospective approach, utilizing existing medical records and data, which may introduce limitations such as incomplete or missing information and potential inaccuracies in data collection. Additionally, the study was conducted at a single centre, Queen Hospital Burton (QHB), which raises concerns regarding the generalizability of the findings to other healthcare settings and populations. The relatively small sample size of 139 patients also warrants consideration, as a larger sample would enhance the robustness and generalizability of the results. Selection bias is another potential limitation, as the study only included patients referred to the Orthopedic team at QHB, possibly resulting in an under-representation of severe cases referred to other departments or hospitals.

Furthermore, the absence of a control group prevents meaningful comparisons and limits the assessment of outcomes and management strategies for chest trauma patients. It is important to note that the study highlights the lack of national guidelines for the management of rib fractures and chest trauma, which may have influenced the observed management approaches and outcomes. Lastly, the study primarily focused on immediate outcomes and the duration of hospital stay, lacking comprehensive long-term follow-up data on patient outcomes, functional recovery, and quality of life.

For future studies, it is recommended to conduct a more comprehensive analysis of patient deaths from earlier stages to gain a deeper understanding of the factors contributing to mortality. Furthermore, it is crucial to review the existing protocol with the ED to ensure efficient and appropriate in-hospital admissions for patients with chest trauma.

## Conclusions

The majority of patients in this study were aged 65 and over and presented with multiple comorbidities, indicating the complex medical profile of this population. Notably, all patients with life-threatening injuries were at a higher risk of complications, regardless of the number of rib fractures sustained. However, despite the presence of life-threatening injuries and the associated risks, only a minority of patients in the study were transferred to a designated trauma centre. This raises concerns about the adequacy of the current transfer protocols and the potential impact on patient outcomes.

## References

[1] Battle C, Hutchings H, Lovett S (2014). Predicting outcomes after blunt chest wall trauma: development and external validation of a new prognostic model. Crit Care.

[2] (2022). Trauma Audit and Research Network: major trauma in older people. https://www.tarn.ac.uk/Content.aspx..

[3] Witt CE, Bulger EM (2017). Comprehensive approach to the management of the patient with multiple rib fractures: a review and introduction of a bundled rib fracture management protocol. Trauma Surg Acute Care Open.

[4] Holcomb JB, McMullin NR, Kozar RA, Lygas MH, Moore FA (2003). Morbidity from rib fractures increases after age. J Am Coll Surg.

[5] Bulger EM, Arneson MA, Mock CN, Jurkovich GJ (2000). Rib fractures in the elderly. J Trauma.

[6] Pieracci FM, Coleman J, Ali-Osman F (2018). A multicenter evaluation of the optimal timing of surgical stabilization of rib fractures. J Trauma Acute Care Surg.

[7] Peek J, Smeeing DP, Hietbrink F, Houwert RM, Marsman M, de Jong MB (2019). Comparison of analgesic interventions for traumatic rib fractures: a systematic review and meta-analysis. Eur J Trauma Emerg Surg.

[8] Unsworth A, Curtis K, Asha SE (2015). Treatments for blunt chest trauma and their impact on patient outcomes and health service delivery. Scand J Trauma Resusc Emerg Med.

[9] Lodhia JV, Konstantinidis K, Papagiannopoulos K (2019). Surgical management of multiple rib fractures/flail chest. J Thorac Dis.

